# The effects of acute exercise on memory: considerations for exercise duration and participant body mass index

**DOI:** 10.1007/s00426-025-02120-5

**Published:** 2025-04-19

**Authors:** Zakary Patrick, Myungjin Jung, Terry McMorris, Paul D. Loprinzi

**Affiliations:** 1 Exercise & Memory Laboratory, Department of Health, Exercise Science and Recreation Management, University of Mississippi, Oxford, MS 38655 USA; 2 Department of Kinesiology, Health Promotion and Recreation, University of North Texas, Denton, TX USA; 3 University of Chichester, College Lane, Chichester, West Sussex UK; 4 Department of Psychology, University of Mississippi, Oxford, MS USA

## Abstract

Acute moderate-intensity exercise has been demonstrated to improve memory performance. It is less clear, however, whether the duration of acute exercise and body mass index (BMI) may moderate this effect. Thus, the purpose of this experiment was to evaluate the effects of differing exercise durations (20- and 40-minutes) on immediate and long-term memory performance, while considering BMI as a moderating factor in this exercise duration and memory performance relationship. Twenty-three young healthy adults participated in a within-subjects experiment. Participants completed four different experimental visits including either exercising at a moderate intensity (or standing on a treadmill) for 20- or 40-minutes, followed by an immediate free-recall memory assessment and then a delayed 24-hr recall. Acute moderate-intensity exercise improved memory performance, regardless of the duration of exercise. Further, long-term memory performance was greater for individuals with a higher BMI when they engaged in shorter (20 min) exercise compared to longer (40 min) exercise.

## Introduction

Since the early 2000s, a vast abundance of literature has suggested that acute moderate-intensity exercise positively influences memory performance (Labban & Etnier, 2011; Loprinzi et al., 2021a; Tomporowski, 2003). This effect can be explained through physiological mechanisms, such as increases in cerebral blood flow and expression of brain-derived neurotrophic factor (BDNF) (Loprinzi et al., 2021), and/or psychological mechanisms like motivation or attention toward encoding/retrieval (Tomporowski & Tinsley, 1996). Increases in BDNF from acute, vigorous intensity exercise have demonstrated short-term learning effects, potentially from BDNF’s involvement in hippocampal long-term potentiation (i.e., synaptic plasticity) (Winter et al., 2007). Moreover, psychological mechanisms, like attention, have been theorized to play an important role in encoding; such that, successful memory encoding relies on the extent to which information is attended, organized, and processed deeply (Richter & Yeung, 2016). Acute exercise, and its purported benefits on cognition, has also been associated with executive function, suggested as a moderating factor in this relationship (Ishihara et al., 2021), as well as a mediator, specifically through facilitated inhibition in children (Drollette et al., 2014).

The observed increase in memory performance due to acute exercise has been widely investigated, ranging from animal models to behavioral experiments in humans (Voss et al., 2013). In addition to the aforementioned mechanisms, another potential underlying mechanism includes the catecholamines hypothesis, which proposes that increased peripheral release of noradrenaline (NA) induces increases in brain NA and dopamine (DA), which benefit cognition (including memory) (McMorris, 2016). This catecholamines hypothesis is the guiding framework for the current experiment.

In addition to the potential mediators, selected moderators that have been further explored within the exercise and memory relationship include the intensity of exercise (Loprinzi et al., 2023) and the duration of exercise (Crush & Loprinzi, 2017; Hacker et al., 2020). Previous work has demonstrated intensity-specific effects on other domains of cognition; acute moderate-intensity exercise suggested to positively influence higher order cognitive function, such as executive function (Chang et al., 2012; Pontifex et al., 2009), whereas acute vigorous-intensity exercise potentially benefitting highly automated behavior (McMorris, 2016), such as reaction time (Chueh et al., 2023). Minimal work has focused on the effects of acute exercise duration on short- and long-term memory, which will be addressed further in subsequent sections and is the primary purpose of this experiment.

### Acute exercise and memory: Intensity-specific effects

Exercise intensity can have a wide range of effects on both physiological and psychological parameters. Previous meta-analyses have reported that acute exercise improves short-term or working memory and long-term memory, with lower intensities exhibiting a greater benefit on working memory (Loprinzi, 2018; Roig et al., 2013). Furthermore, acute vigorous-intensity exercise immediately before encoding has been shown to improve episodic memory performance (Loprinzi et al., 2019). Under the framework of the catecholamines hypothesis, vigorous-intensity exercise, an intensity beyond the necessary catecholamine threshold, may temporarily increase levels of NA (Loprinzi, 2018). In addition to elevating circulating catecholamines via exercise intensity, the duration of exercise may modulate the concentration of NA (McMorris, 2021).

### Acute exercise and memory: Duration-specific effects

The length of acute exercise duration and its association with memory performance has received less attention. Crush and Loprinzi (2017) aimed to elucidate the potential relationship between exercise duration and cognition in young adults across different conditions implementing bouts of exercise of 10-, 20-, 30-, 45-, and 60-minute duration, but did not observe consistent evidence of any duration-specific effects on cognition. Yet, the cognitive tasks (Trail Making Tests A and B assessing visual attention, visual motor speed, and set switching; spatial span memory task; Stroop color word test) did not explicitly include either a memory-recognition or memory-recall assessment. Further on this theme in a young adult population, Hacker et al. (2020) investigated the effects of acute exercise duration on visual recognition and attention performance, determining that exercise duration elicited conflicting outcomes; other than positive influences on arousal, cognitive outcomes seemed to be positive for 15-minute durations and negative for longer durations. Outside of these aforementioned papers, a meta-analysis by Roig et al. (2013) investigating the potential mechanisms underlying the exercise and memory relationship performed a sub-group analysis evaluating the effects of exercise duration on short- and long-term memory. Their meta-analysis found that acute exercise of short (i.e., < 20-min) and medium (i.e., 20-40-min) duration had the largest effects on long-term memory; however, this sub-group analysis included studies implementing various exercise durations. Additionally, Chang et al. (2012) conducted a meta-analysis that evaluated numerous moderators of the acute exercise-cognition interaction. Regarding acute exercise duration, they demonstrated that longer durations (20 + min) improved cognitive performance. To our knowledge, there are no other published experiments specifically investigating the association between acute exercise duration and long-term memory performance, as initial work has only investigated duration and procedural memory in children (Angulo-Barroso et al., 2019) and duration and executive function (Chang et al., 2019; Hacker et al., 2020). Thus, the lack of investigation and information serves as the foundation for the primary aim of this study.

### Acute exercise and memory: the catecholamines hypothesis as a potential basis for duration-specific effects on memory performance

With an emphasis on the explorations of exercise duration as a potential influence on memory performance, a potential physiological explanation may be the increase in circulating catecholamines due to moderate-intensity exercise. McMorris (2016) suggested that NA may play an important role in the exercise-memory interaction, deemed the catecholamines hypothesis. Among other potential mechanisms (discussed above) that might explain the observed increases in memory performance due to exercise, increases in peripherally circulating NA, stimulated by short-to-moderate duration of exercise, may activate vagal afferents that relay information to the nucleus tractus solitarii, which projects to the locus coeruleus and stimulates NA release in the brain (McMorris, 2016). Acute exercise has also been hypothesized to induce effects on neuronal excitability through muscle spindle activity, leading to action potential generation and propagation through the dorsal spinal cord to the nucleus tractus solitarii, and, ultimately the locus coeruleus, which has projections to brain structures that subserve memory function (Loprinzi et al., 2018). Previous methodological evaluations of the catecholamine response to exercise have demonstrated the aforementioned intensity-specific increases in NA (Dimsdale et al., 1984).

McMorris followed up this original work with a more thorough review of the catecholamines hypothesis, suggesting that exercise duration may be sufficient to increase NA concentrations even when engaging in exercise that is not vigorous (Christensen et al., 1979; Green et al., 1983; McMorris, 2021). This exercise duration-specific increase in NA is postulated to be a result of an increase in tonic releases as moderate-intensity exercise progresses toward central fatigue, albeit direct empirical evidence is limited (Hodgetts et al., 1991). Regarding the progression to central fatigue, this increase, and quickening, of the tonic release of locus-coeruleus NA (Aston-Jones et al., 2000) may subserve learning and long-term memory specific tasks (Aston-Jones & Cohen, 2005). Thus, differing durations of acute exercise may influence memory performance via the catecholamines hypothesis.

### Present experiment

The present experiment sought to evaluate the potential memory benefits as a result of differing exercise durations. Importantly, our aim is not to empirically test the catecholamine hypothesis (i.e., measure NA as a mediator), but rather, to simply use this framework as a justification for why we think there could be memory differences based on the duration of acute exercise. By systematically manipulating the duration of the respective experimental conditions (exercise v. control/rest), the primary objective of the present experiment was to evaluate the acute effects of moderate-intensity exercise duration on memory performance.

An exploratory objective of the study was to evaluate whether a relevant moderator may be associated with duration-specific effects from acute exercise, with previous literature informing this secondary aim focused on body mass index (BMI). Obesity has been associated with reduced neural activity in the hippocampus (Cheke et al., 2017). Recent research has reported on the effects of bariatric surgery and the positive changes on cognition (Custers et al., 2024; Vreeken et al., 2023). A recent review concluded an inverse association between obesity and memory performance (Loprinzi & Frith, 2018). Additionally, a review (Chang et al., 2017) explored the available literature incorporating variables of physical activity, cognition, and obesity; the authors report conflicting results of obesity as a moderating factor in the exercise and cognition relationship. Thus, these conflicting results warrant further investigation. For this exploratory secondary aim, the available data for the variable of obesity was BMI.

For the primary objective, memory performance was assessed using free recall, which may be more sensitive to acute exercise when compared to other types of memory assessments, such as cued recall and recognition (Moutoussamy et al., 2022). Assessing long-term memory, we employed two separate free recall memory assessments at 20-minutes and 24-hours after encoding. With prior work suggesting that less intense exercise (e.g., not vigorous-intensity) may increase NA if lasting at least 30 min (McMorris, 2021), our experimental protocol specifically implemented exercise durations of 20- and 40-minutes. Per the predictions of the catecholamines hypothesis, moderate-intensity exercise durations greater than 30-minutes should acutely elicit a greater memory benefit than the 20-minute exercise condition. Based on this prediction, we predicted that the longer duration condition (40-min) of moderate-intensity exercise would show greater long-term memory performance than the shorter exercise duration (20-min), with both of these exercise conditions improving memory performance compared to the control (no exercise) condition. Thus, we expected to observe a main effect of condition (exercise is better than control), but then also a condition (exercise v control) by duration (20 min of exercise/control v 40 min of exercise/control) interaction. Regarding our exploratory objective and based on prior literature and some previous unpublished work in our lab, it is possible that BMI may interact with acute exercise to influence memory. However, we reserve caution in rendering strong predictions, as this was an exploratory analysis in which the study was not a-priori designed to evaluate.

## Methods

### Participants

Twenty-three University of Mississippi students (17.4% female; 18–25 years) took part in the study. Participants were excluded if they (1) self-reported as a daily smoker; (2) self-reported being pregnant; (3) exercised within 5 h of testing; (4) consumed caffeine within 6 h of testing; (5) took medications used to regulate emotion (e.g., SSRI’s); (6) had a concussion or head trauma within the past 12 months; (7) took marijuana or other mind-altering drugs within the past 2 days; (8) were considered a daily alcohol user (> 30 drinks/month for women; > 60 drinks/month for men) or consumed alcohol in the past 12 h; or (9) answered “yes” to any of the questions on the PAR-Q (Physical Activity Readiness Questionnaire).

### Study design and procedures

**Fig. 1:**
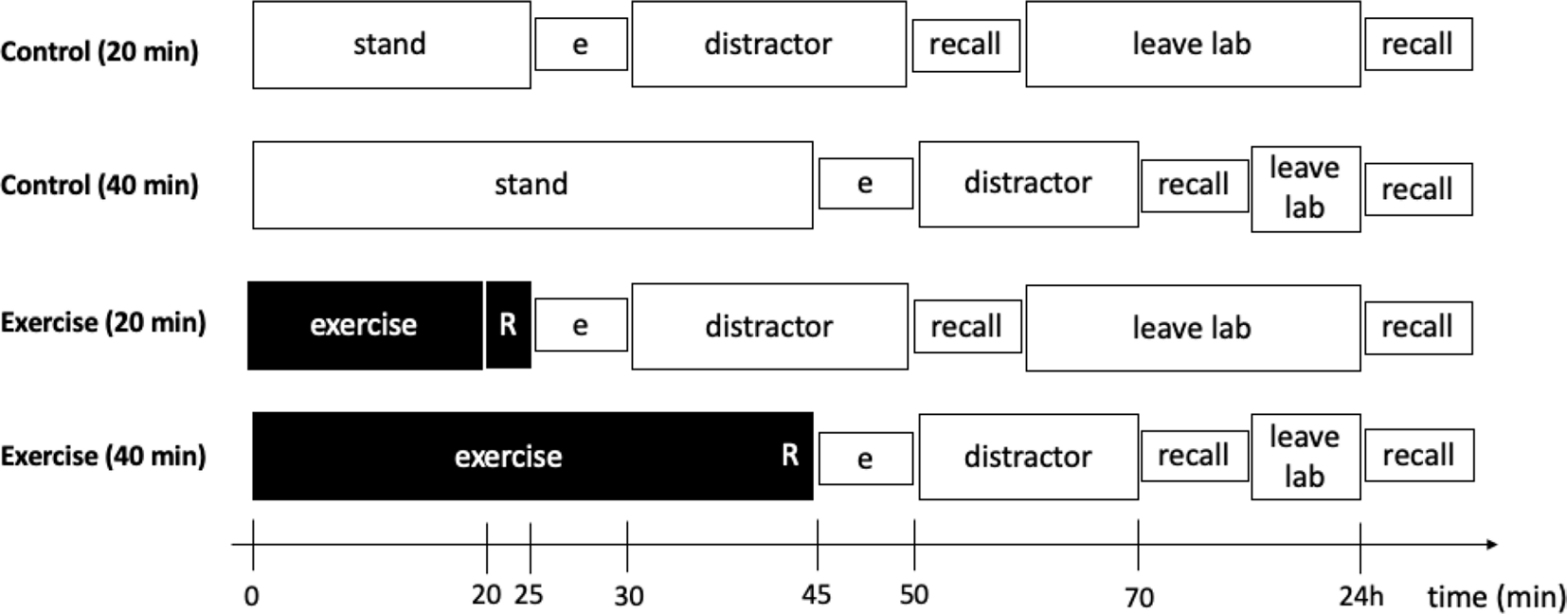
The four experimental conditions encompassed 8 visits. Each participant completed the four conditions, which included the experimental visits of control or exercise across either 20-min or 40-min acute exercise durations, with each experimental visit followed by a 24-hour visit for the long-term memory recall assessment. R = rest after the respective exercise condition; e = encoding phase

A 2 (*Condition*: Control v Exercise) × 2 (*Duration*: 20 min v 40 min) × 2 (*Memory Assessment*: 20-min Delay, 24-hr Delay) factorial design was employed. All factors occurred as within-subject factors. Allocation concealment occurred by both the researcher and participant not knowing which condition they would complete until arriving in the lab. Randomization was performed using a computer-generated algorithm. Participants completed 9 visits in total. The first visit included a maximal exercise (treadmill) test to determine the participant’s maximal heart rate (HR) and to also familiarize the participant with the memory protocol. The maximal HR achieved during the first visit was used to set the personalized exercise intensity. After the initial visit, eight subsequent visits occurred, as noted below. See Fig. 1 for a schematic of the study procedures. The standard for all follow-up experimental condition visits (i.e., after the 24-hour recall of the previous experimental visit) was 24–72 h. All experimental procedures were approved by the University of Mississippi Institutional Review Board, and participants provided written informed consent before participation.

### Maximal exercise visit (1st session)

The first laboratory visit included a maximal treadmill-based assessment. The specific assessment included an individualized protocol (Loprinzi et al., 2023). Participants performed a warm-up for 3-min by walking at 3.5 miles per hour (mph). Following this, they engaged in a constant speed throughout the test while the grade increased by 2% every 2-minutes. After the warm-up period, the speed was set, and remained, at 5.5 mph for the entire exercise protocol. The duration (min: sec) was used as a measure of endurance performance (proxy for cardiorespiratory fitness). Notably, time-to-exhaustion protocols, such as this, are highly correlated with direct measures of oxygen consumption (*r* =.91-0.94) (Pollock et al., 1982). During the maximal treadmill test, HR was monitored throughout the test. Rating of perceived exertion (RPE) was evaluated (a 6–20 scale that is used to determine the participants’ perception of exertion during exercise) at the conclusion of the bout of exercise (Borg, 1982). The maximal treadmill exercise bout ended when the participant elected to stop exercising.

### Control and Moderate-Intensity exercise visits

Participants completed two Control conditions, involving watching a video (self-selected video, either The Office or The Big Bang Theory) for either 25–45 min. Following this, they completed a memory task. The two exercise visits involved either exercising for 20 min (with 5-minute recovery period) or 40 min (with 5-minute recovery period). Following this, they completed a memory task. Twenty-four hours after completing the visit, they returned to the lab to recomplete a memory recall task.

During the exercise visits, based on the participant’s maximal HR achieved during the first session, they exercised at 50% of their heart rate reserve (HRR) (Garber et al., 2011). Heart rate reserve was calculated as ([HR_max_– HR_rest_) * % target intensity] + HR_rest_). The resting HR measurement was recorded after sitting quietly for five minutes^1^. This moderate intensity of 50% of HRR is well above the intensity of prior work (e.g., 30% of VO_2_ max) that has been shown to be effective in elevating levels of NA (Garber et al., 2011), and was selected to ensure participants could appropriately complete both exercise conditions of 20- and 40-minute durations. Notably, all participants were able to complete all exercise conditions.

### Memory assessment. Study list

The memory task was programmed in E-Prime (v.3). Similar to common list-learning paradigms (e.g., Ray Auditory Verbal Learning Test), participants were exposed to 5 trials, each including 15 words. The words were identical across the 5 trials, but were displayed in a random order, across trials and participants. Separate word lists were used for each of the conditions, and ordering of the word lists was randomized across the counterbalanced visit order. Each word remained on the screen for 1500 milliseconds and participants said the word aloud as it appeared (to ensure they are processing the stimuli). Notably, this memory protocol has been shown to be sensitive to exercise-related improvements in memory (Loprinzi et al., 2021).

Each word list was created by utilizing the MRC Psycholinguistic Database from the University of Western Australia. The following criteria was set for each word: number of letters (4–10), number of syllables (1–3), familiarity rating (450–700), concreteness rating (450–700), imageability rating (450–700), meaningfulness rating (450–700), and only nouns were used. No two words within each list were semantically related (*r* <.30). Further, a similar proportion of animate words (i.e., animates can act; grow and reproduce; know, perceive, emote, learn and deduce; and made of biological structures that maintain life) appear in each list (Bonin et al., 2014). (An ANOVA demonstrated that, across the four lists, there was not a statistically significant difference for the number of letters, *F*(3, 59) = 0.12, *p* =.94, *M* (SE) = 5.96 (0.16), number of syllables, *F*(3, 59) = 0.10, *p* =.96, *M* (SE) = 1.7 (0.08), familiarity rating, *F*(3, 59) = 0.49, *p* =.68, *M* (SE) = 538.7 (9.6), concreteness rating, *F*(3, 59) = 0.74, *p* =.53, *M* (SE) = 555.9 (6.5), imageability rating, *F*(3, 59) = 1.34, *p* =.27, *M* (SE) = 567.2 (5.9), meaningfulness rating, *F*(3, 59) = 0.30, *p* =.82, *M* (SE) = 491.8 (4.6), and proportion of animate words, *F*(3, 59) = 0.35, *p* =.78, *M* (SE) = 0.23 (0.05).

### Recall assessment

Immediately after encoding the 5th trial, participants watched a self-selected video (The Office or The Big Bang Theory) for 20 min (no sound on the video; low arousal consolidation period). While viewing the video, participants were asked to draw 3 small pictures that depict 3 major scenes from the 20-minute video; this was implemented to avoid participants rehearsing the words during this 20-minute delay period. After this 20-minute delay period, participants freely recalled as many words as possible. 20-minute delay period was selected as past work demonstrates that this delay period is sensitive to exercise-induced improvements in memory function (Loprinzi et al., 2020). Following this 20-minute delayed recall assessment, participants left the lab. Participants then returned 24-hours later for a long-term free recall assessment. For both recall assessments (20-min and 24-hr delay), after the participant recalled their final word, they were encouraged to try and recall at least one more word; this was implemented to avoid minimal effort during memory recall. For each recall assessment, the participant was given 3 min to recall the items from the respective list. The subsequent laboratory visit did not occur until after the 24-hour free recall assessment had occurred. For the recall assessments, two outcomes were calculated, namely the number of correctly recalled words for that condition and the number of incorrectly recalled words (i.e., recalling a word not on the list for that condition, which could include a word from no lists or an intrusion from another list). All word lists used in the paradigm were counterbalanced across conditions.

### Analyses

Our primary aim was to evaluate if there is a duration-specific effect of acute moderate-intensity exercise on memory recall performance. To investigate this primary aim, a 2 (*Duration*: 20 min, 40 min) × 2 (*Condition*: Control, Exercise) linear mixed model analysis was employed. Models were computed separately for the different memory outcomes, namely 20-min delayed true recall (# of correct words), 24-hour delayed true recall (# of correct words), 20-min delayed false recall (# of incorrect words), and 24-hour delayed false recall (# of incorrect words).

Our exploratory aim, by utilizing linear mixed model analyses, was to evaluate whether BMI interacted with moderate-intensity exercise duration to influence memory performance. To investigate this aim, a 2 (*Condition*) × 2 (*Duration*) linear mixed model was computed for the same aforementioned dependent variables but utilizing separate models for BMI as a potential moderator.

All linear mixed model analyses were computed in SPSS (v 29) using general guidelines (West, 2009). Subject ID was a nominal variable that served as a random effects variable with the inclusion of the intercept; Condition (two-level ordinal variable) and Duration (two-level ordinal variable) served as fixed-effects variables with the theoretical moderators entered into the models as covariates (Field, 2015) for the secondary aim. The estimation method used was Maximum Likelihood (ML). For the fixed effects, Type III tests were used for the sum of squares estimation. Degrees of freedom were estimated using Satterthwaite approximation. Linear mixed modeling was chosen for this analysis to prevent having to exclude select participants with missing data, as well as to account for individual differences in memory performance.

Based on a sensitivity analysis, with inputs of an α of 0.05, power of 0.80, 23 participants, 4 conditions (2 × 2 design), and an assumed repeated measures correlation of 0.50, we had sufficient power to detect a medium effect (effect size f of 0.25). This 23-participant sample is similar to previous publications investigating the exercise and memory relationship in this population (Chang et al., 2015; Slutsky et al., 2017; Slutsky-Ganesh et al., 2020).

Data, along with the syntax code for computational reproducibility, can be found at: https://osf.io/ws4hr/.

## Results

### Participant characteristics

Table 1 displays the demographic and behavioral characteristics of the sample. The participants, on average, were 19.7 years old (SE = 0.35; range = 18–24), with the sample containing 82.6% male participants. Participants were physically active, well above American college o (mean of 226.1 min/week of moderate-to-vigorous intensity physical activity engagement^2^).

**Table 1:** Demographic, behavioral, and performance characteristics of the sample (*n* = 23)

Variable	Point Estimate (Range)	SE
Age, mean years	19.7 (18–24)	0.35
Sex, % Male	82.6	
Measured body mass index, mean kg/m^2^	25.1 (19.3–35.6)	0.87
Physical activity, mean min/week of MVPA	226.1 (0-500)	30.12
Duration lasted on maximal treadmill test, mean seconds RPE at end of maximal treadmill test, Borg scale	911.1 (228–1496) 18.4 (15–20)	58.49 0.29

Note MVPA = Moderate-to-vigorous intensity physical activity

**Fig. 2:**
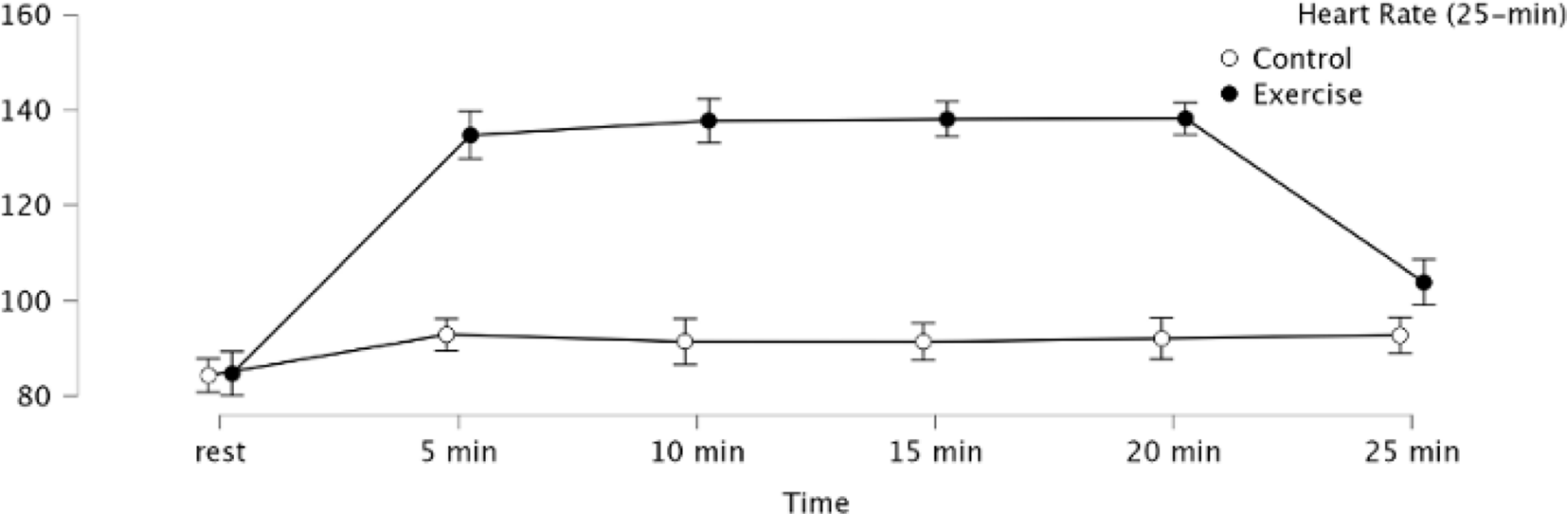
Heart rate values among the two 25-min duration experimental conditions. Error bars represent 95% confidence intervals (CI)

**Fig. 3:**
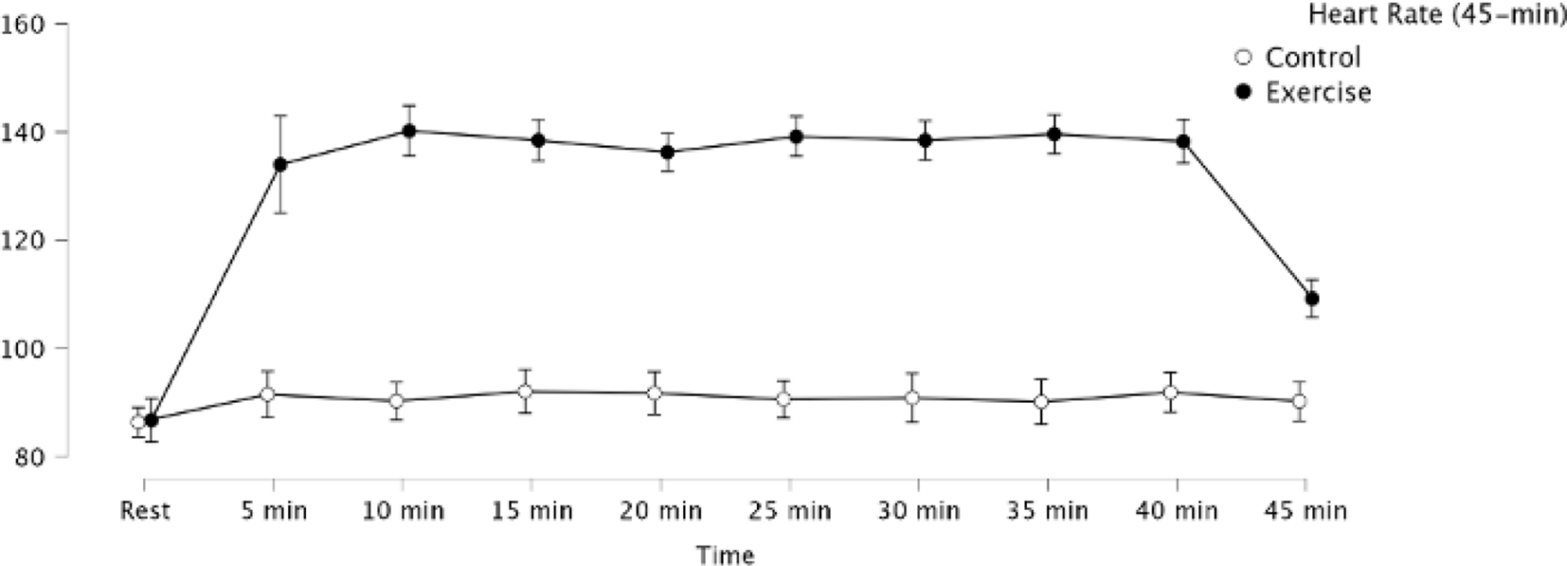
Heart rate values among the two 45-min duration experimental conditions. Error bars represent 95% CI

Target HR for the two exercise conditions were 50% HRR, corresponding to a 138.6 bpm in the present sample. Across the two moderate-intensity exercise conditions (25-minute and 45-minute conditions, including the 5-minute rest period), the mean HRs at the endpoints of exercise (20-min and 40-min, respectively) were 138.2 and 138.3, respectively. Figures 2 and 3 show the HR responses across the 25- and 45-minute periods for the exercise and control conditions.

### Memory performance

Table 2 displays the memory results (Words Correct 20-min delay, Words Incorrect 20-min delay, Words Correct 24-hr delay, Words Incorrect 24-hr delay) across the conditions.

With memory performance for Words Correct (i.e., true memory) at the 20-min delay as the outcome, a 2 (*Condition*) × 2 (*Duration*) linear mixed model analysis demonstrated that the Type III tests of fixed effects yielded a main effect for Condition, *F*(1, 84) = 4.415, *p* =.04, but no main effect for Duration, *F*(1, 84) = 1.013, *p* =.32, and no interaction of these factors, *F*(1, 84) = 0.003, *p* =.95. For the significant main effect of Condition, the exercise group recalled more words than the control group, M_diff_ = 0.75, SE = 0.36, *p* =.04. Regarding the number of incorrect words (i.e., false memory) serving as the outcome at the 20-min delay, the type III fixed effects yielded no main effects for Condition *F*(1, 84) = 0.02, *p* =.90, Duration, *F*(1, 84) = 1.07, *p* =.30, or an interaction of these factors, *F*(1, 84) = 0.04, *p* =.85. The results suggest that the exercise condition, regardless of duration, improved memory performance at the 20-minute delay memory recall when compared to the non-exercise control condition.

With memory performance for Words Correct (i.e., true memory) at the 24-hour delay, the Type III fixed effects yielded a main effect of Condition, *F*(1, 83) = 6.51, *p* =.01, but no main effect for Duration, *F*(1, 83) = 0.29, *p* =.59, and no interaction of these factors, *F*(1, 83) = 1.40, *p* =.24. For the significant main effect of Condition, the exercise group recalled more words than the control group, M_diff_ = 1.07, SE = 0.42, *p* =.01. These findings suggest that exercise improved memory performance at the 24-hour delay when compared to the non-exercise control condition. At the same 24-hour delay for Words Incorrect (i.e., false memory) as the outcome, the Type III test of fixed effects yielded no main effects for Condition, *F*(1, 83) = 0.04, *p* =.84, or Duration, *F*(1, 83) = 2.42, *p* =.12, but did yield an interaction of these factors, *F*(1, 83) = 4.45, *p* =.04. Post-hoc decomposition of the Condition and Duration interaction using LSD (Fisher’s Least Significant Difference) method determined a significant comparison between Exercise 20-min duration and Exercise 40-min duration, *p* =.013 (Bonferroni adjusted *p* =.013), with the Exercise 20-min condition having a greater (M = 1.465, SD = 0.260) number of incorrectly words recalled than the Exercise 40-min condition (M = 0.659, SD = 0.271). These results suggest that the 20-min exercise condition falsely recalled more words than the condition in which the participants engaged in 40-minutes of moderate intensity exercise.

**Table 2:** Memory results (estimated marginal means) (SE) across conditions

Condition	20-min delay Correct	20-min delay Incorrect	24-hr delay Correct	24-hr delay Incorrect
Control (20 min) and 5 min rest	8.04 (0.75) (*n* = 23)	0.91 (0.27) (*n* = 23)	6.57 (0.75) (*n* = 23)	0.96 (0.25) (*n* = 23)
Control (40 min) and 5 min rest	8.42 (0.76) (*n* = 22)	0.66 (0.27) (*n* = 22)	6.30 (0.75) (*n* = 22)	1.08 (0.26) (*n* = 22)
Exercise (20 min) and 5 min rest	8.81 (0.76) (*n* = 22)	0.85 (0.27) (*n* = 22)	7.14 (0.75) (*n* = 22)	1.47 (0.26) (*n* = 22)
Exercise (40 min) and 5 min rest	9.15 (0.76) (*n* = 21)	0.67 (0.28) (*n* = 21)	7.86 (0.77) (*n* = 20)	0.66 (0.27) (*n* = 20)

**Fig. 4:**
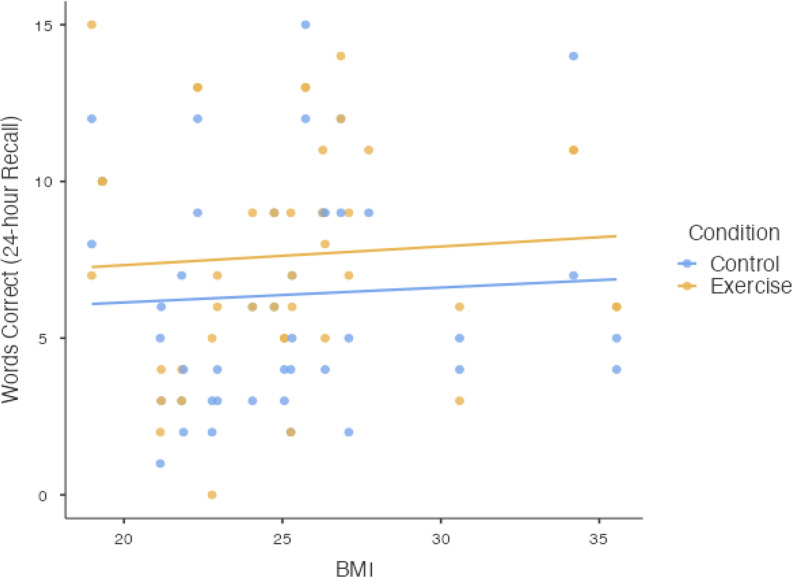
Scatterplot depicting results for Words Correct at the 24-hour Recall on the ordinate with BMI values of the sample on the abscissa, with separate lines representing the different experimental conditions (Control v. Exercise) collapsed across the factor of Duration

**Fig. 5:**
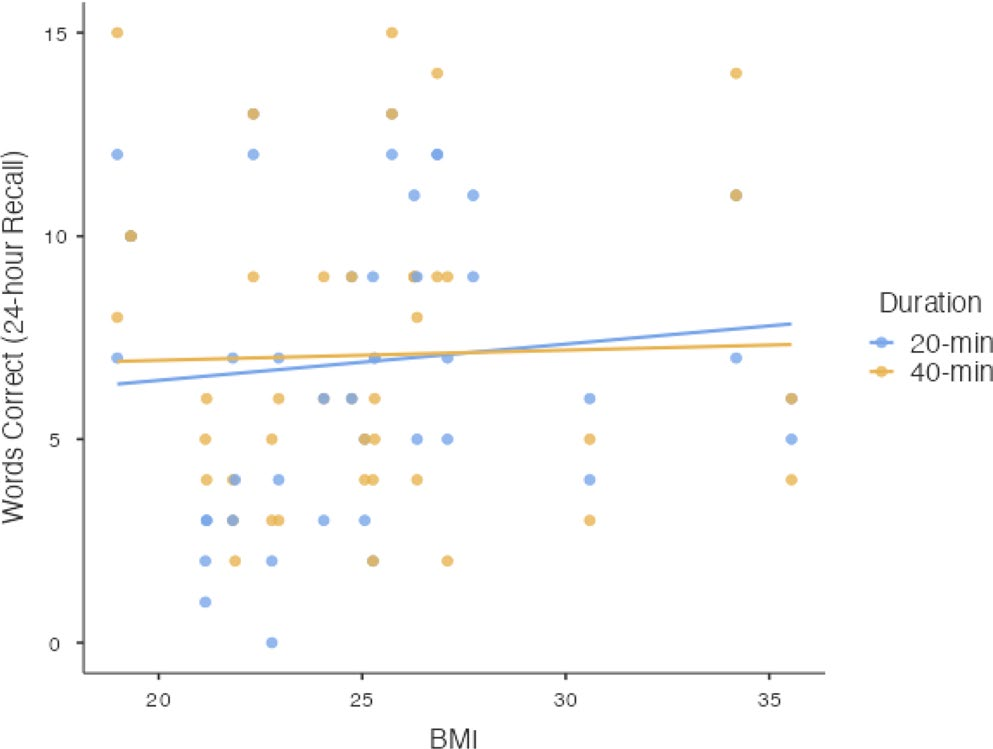
Scatterplot depicting results for Words Correct at the 24-hour Recall on the ordinate with BMI values of the sample on the abscissa, with separate lines representing the different experimental manipulations of exercise duration (20-min v. 40-min) collapsed across the factor of Condition

To evaluate the potentially relevant moderator of BMI, this covariate was included as a fixed factor for each of the respective memory performance outcomes^3^. With Words Correct at the 24-hour delay as the outcome, the Type III fixed effects yielded a 3-way interaction of Condition × Duration × BMI, *F*(1, 79) = 4.84, *p* =.03. To decompose this 3-way interaction, we plotted memory performance (24-hour recall) for the two conditions (exercise v control) across levels of BMI (see Fig. 4), and also plotted memory performance for the two levels of exercise duration (20 min v 40 min) across levels of BMI (see Fig. 5).

As shown in Fig. 4, memory recall at the 24-hour delay is represented on the y-axis and BMI (kg/m^2^) values are represented on the x-axis. With the plotted lines representing each level of Condition collapsed across the fixed factor of Duration, regardless of BMI, participants performed better on the long-term memory assessment after the exercise condition when compared to the long-term memory assessment after the control condition.

As shown in Fig. 5, the memory performance at the 24-hour delay is represented on the y-axis and the participants’ BMI values (kg/m^2^) are represented on the x-axis. The two plotted lines represent the two levels of the fixed factor of Duration collapsed across the fixed factor of Condition. Results showed that memory performance was higher for the longer exercise duration in participants with a lower BMI when compared to the shorter exercise duration, but as BMI increased, participants with a higher BMI demonstrated reduced memory performance in the longer exercise condition; importantly, the memory performance was worse than the shorter exercise condition.

## Discussion

The primary purpose of the present experiment was evaluating, via direct manipulation of the exercise duration, whether there are any duration-specific effects of acute moderate-intensity exercise on memory performance. An exploratory, secondary objective was to evaluate if BMI influenced this relationship. Although the results of the current experiment are in line with previous studies regarding the beneficial effect of acute exercise on memory recall performance using other modalities, durations, and intensities (Roig et al., 2013; Chang et al., 2012; Loprinzi et al., 2019), memory performance did not differ between the two experimental manipulations of exercise duration. As for the exploratory analyses, there was a significant three-way interaction between condition, duration, and BMI, such that, for example, memory performance was greater for individuals with a higher BMI when they engaged in shorter (20 min) acute exercise compared to longer (40 min) exercise.

### Main findings

One of the notable findings of the present experiment was that moderate-intensity acute exercise was effective in enhancing long-term episodic memory performance. Encouragingly, we observed this effect for both the 20-min and 24-hr delayed memory assessments. These findings support other work demonstrating that an acute bout of moderate-intensity exercise can have long-lasting effects on memory (Labban & Etnier, 2011; Loprinzi et al., 2021a). To extend these exciting findings, we evaluated whether varying the acute exercise duration would have a unique effect on improvements in memory from exercise, which allowed us to indirectly test the catecholamines hypothesis suggested by McMorris (2016, 2021).

The catecholamines hypothesis suggests that a larger physiological response, such as a longer exercise duration, should increase select neurotransmitters (e.g., NA and DA) to improve memory performance. To test this theory, we evaluated whether memory performance would vary as a function of the duration of acute exercise, 20-min versus 40-min. Initially, we predicted that this would be an adequate comparison to evaluate the catecholamines hypothesis, as prior work demonstrates that substantive increases in NA occur after 30 min of moderate-intensity exercise (McMorris, 2021). Our lack of a difference in memory performance between 20- and 40-minutes of acute exercise may be a result of several factors. First, our sample was very active (USDHHS, 2018). Perhaps a 40-min bout of moderate-intensity acute exercise was not enough of a difference (when compared to 20-min) to observe meaningful differences in arousal and NA concentrations. Second, per the catecholamines hypothesis, NA elevation should enhance the recall of episodic memory via its plasticity-related alterations (e.g., long-term potentiation) (Maity et al., 2020). We intentionally positioned the exercise to occur prior to encoding given that past work demonstrates this to be effective in enhancing memory (Loprinzi et al., 2023). However, other work also shows that positioning the bout of exercise in the early consolidation window is also effective in enhancing long-term episodic memory (Loprinzi, Day, Loprinzi et al., 2021a, b). It is possible that exercising during the early consolidation window may result in greater plasticity-related alterations, as this may be a more ideal window to induce synaptic plasticity given that, after encoding, the neural trace that represents the memory may be in a labile state susceptible to re-consolidation. Based on this, a longer exercise duration (40-min v 20-min) during the early consolidation window may allow for a more robust stimulus to maximize synaptic plasticity to augment memory performance. Separate from episodic memory considerations, recent work has also suggested that acute exercise effects on executive function may be sensitive to differing exercise durations (Chang et al., 2019); similarly, acute exercise duration may also have unique effects on procedural memory (Angulo-Barroso et al., 2019). As the current paper methodologically explored the effects of differing exercise durations on long-term memory performance, the paucity of work directly exploring this research question warrants further investigation into duration-specific effects.

Third, to observe an acceptable progression toward central fatigue, the exercise duration could be increased beyond 40-minutes. Relatedly, perhaps the current exercise protocol at moderate-intensity was not sufficient to increase NA concentration enough to facilitate memory benefits. Alternatively, rather than exercise duration, higher exercise intensity may be the ultimate factor in progressing toward central fatigue and reaping the benefits of increased NA concentration subserving enhanced episodic memory function. Nonetheless, previous work has demonstrated that an exercise intensity as low as 30% VO_2_ max accompanying an exercise duration beyond 30-minutes is sufficient in increasing levels of NA (Hodgetts et al., 1991; McMorris, 2021). Moreover, this elevated circulation of NA has major implications on the functioning of the hippocampus, the integral brain area for learning and memory. However, we did not observe any duration-specific differences on memory performance. Nonetheless, any additional factors could not be stated without considering the warrant for future work to indirectly measure brain catecholamines (using pupillometry or measuring NA and DA metabolites), while undergoing different experimental exercise duration conditions. More recent work has investigated locus-coeruleus activation following acute exercise, reporting that pupillometry changes indicative of NA release were not associated with moderate intensity exercise; however, a neuroelectric index of NA release, the P300 component, was associated with the bout of moderate intensity exercise (McGowan et al., 2019). Thus, further work is warranted.

Fourth, we were only powered to detect a medium effect size. The number of words recalled at the 24-hr delay was 7.1 (out of 15) in the 20-min exercise condition and 7.9 in the 40-min exercise condition.^4^ This magnitude of difference has previously been found to be statistically significant in other work that had a sample size that was 2–3 times higher than the present study (Loprinzi et al., 2023). Thus, although the current data does not support the catecholamines hypothesis based on the level of statistical significance alone, the numeric values (7.1 v 7.9 words) suggest otherwise. Thus, future work on this topic may require a larger sample to observe memory differences when comparing two exercise conditions that both improve memory (relative to control) and are likely to only be subtly different.

### Exploratory finding: BMI

The secondary objective of the current experiment presented an interesting finding: memory performance was higher for the longer exercise duration in participants with a lower BMI when compared to the shorter exercise duration, but as BMI increased, participants with a higher BMI demonstrated reduced memory performance in the longer exercise condition; such that, those with a higher BMI benefitted more from the shorter exercise duration (i.e., 20 min) compared to their higher BMI counterparts. With previous work reporting conflicting results of BMI moderating this relationship (Chang et al., 2017), this observation may suggest an increased consideration for BMI as a relevant moderator in future research, as it may influence exercise and memory with regard to duration and intensity.

BMI may be a valuable consideration as obesity may affect the exercise-cognition relationship via leptin resistance; animal model work demonstrated that obesity impairs the consolidation pathways of memory (Zanini et al., 2017). Furthermore, increases in cytokine production resulting from obesity have been associated with decreased cognitive performance (Yirmiya & Goshen, 2011). In considering these findings in the context of our results and the predictions of the catecholamines hypothesis, we wonder whether factors associated with BMI (or obesity) may alter the catecholamine effects of exercise on memory. Our exploratory results showed that, for example, longer durations of exercise (40 v 20 min) were only beneficial in enhancing memory among those with a lower BMI. Perhaps the higher cytokine production associated with obesity may counteract the NA-induced consolidation mechanisms of long-term episodic memory (Bourgognon & Cavanagh, 2020). From a psychological perspective, it is also possible that longer durations of acute exercise among those with a higher BMI may result in a negative affective response, ultimately impairing encoding-related processes during the study phase of the experiment. It would be interesting for future research to assess participants’ feelings during exercise to better understand the interaction between affective responses and body composition on the impact of acute exercise duration on memory performance.

### Strengths and limitations

Other limitations regarding the current experiment should be stated. The small sample size (*n* = 23), although acknowledged to be suitable for investigating the effects of acute exercise and memory performance, and use of a convenience sampling approach do not allow for suitable generalizability to the greater population. Further, as stated above, this may have prevented our ability to detect a small effect that is likely to occur when comparing memory differences between two exercise conditions. Furthermore, the sample was heavily male (~ 83%) which may also affect the interpretation of results. Although not conclusive, there is some suggestive evidence that acute exercise may enhance memory slightly more for females (Johnson & Loprinzi, 2019). Further, meta-analytic work demonstrates that the greatest effect of acute exercise on memory occurs when the sample is (more equally) mixed across males and females (Loprinzi et al., 2019). Lastly, regarding our exploratory analyses evaluating BMI, the use of BMI is not a perfect measure of obesity; thus, future evaluations including skinfold measurements and use of equipment such as Dual X-ray Absorptiometry (DXA) machines could prove to strengthen these initial findings. Additional methodological considerations could be related to the participants’ behavior between the two recall assessments (i.e., sleep the night before the 24-hour recall or exercise engagement between the two memory assessments) as this was not controlled^5^. Finally, study design has been considered a moderator when evaluating the effects of acute exercise on memory, with previous meta-analyses demonstrating mixed findings when comparing between-subjects and within-subjects designs (Chang et al., 2012; Qazi et al., 2024); thus, future directions can also implement between-subjects designs to evaluate this relationship, expanding beyond our within-subjects paradigm. Despite these limitations, the current experiment has several notable strengths. First, for the experimental manipulations regarding acute moderate-intensity exercise, the use of a maximal exercise test to individualize intensity for the differing exercise durations across the sample is notable. It also evaluates the moderating effect of BMI on the relationship between acute exercise and memory. Furthermore, the results of this current experiment emphasize the observation that 20-minutes of moderate intensity exercise is sufficient for benefitting memory performance, which may have practical meaningfulness for individual’s interested in incurring some memory benefit from acute, moderate-intensity exercise.

## Conclusion

In conclusion, our findings suggest that differing exercise durations, specifically a 40-minute bout of moderate-intensity exercise, is not a factor in enhancing memory performance beyond a 20-minute condition. This has important time-saving implications for individuals who may have limited time to exercise. Future research should consider exploring different levels of moderate-intensity exercise, increasing exercise intensity at the same longer-duration, or potentially investigating the effects that exercise durations beyond 40-minutes may have on memory performance. Future research should carefully consider BMI as a moderator to further expand on the current findings of this work. The catecholamines hypothesis, related to the duration of acute moderate-intensity exercise, may be a key mechanism in the exercise and memory relationship; more robust approaches to investigating this framework are necessary for future research.

## Data Availability

Data is available within the manuscript.

## Footnotes

1 If resting heart rate was more than five bpm higher on a subsequent visit, participants were asked to sit quietly for an additional 5 min.

2 MVPA was measured via the Physical Activity Vital Sign, which consists of a two-item survey assessing days per week of MVPA and how many minutes of MVPA within those self-reported days of the week (Kuntz, 2021).

3 With BMI included as a moderator in each of the 4 linear mixed models, only the outcome of Words Correct at the 24-hr memory assessment yielded a statistically significant 3-way interaction.

4 Utilizing a similar memory encoding paradigm, the recall performance of the current study is similar to those published previously (Loprinzi et al., 2023).

5 However, these variables were collected at the time of the second memory assessment, and they did not influence the reported outcomes herein.
